# Through the Magical Pink Walkway: A Behavior Setting’s Invitation to Embodied Sense-Makers

**DOI:** 10.3389/fpsyg.2020.01576

**Published:** 2020-09-10

**Authors:** Simon Harrison

**Affiliations:** Department of English, City University of Hong Kong, Kowloon, Hong Kong

**Keywords:** behavior setting, the enactive approach, sense-making, embodied interaction, gesture, affordances, cosmetics

## Abstract

This paper examines an intersection between ecological psychology and the enactive approach brought about by studying sense-making in relation to a behavior setting in Hong Kong and adopting a focus on embodied action and gesture. A cosmetics pop-up store embedded in a downtown shopping mall provides the basis for a case study involving a two-pronged analysis. I first use Barker’s behavior setting theory to describe the publicly accessible structure and dynamics of the store, which reveals a bounded spatiotemporal entity with several interdependent behavior–milieu parts. I then analyze video recordings of my research participant encountering, entering, and exploring this environment. Following an enactive-informed micro-ethnographic approach to embodied communication, I examine her movements, postures, gestures, and language use as she joins the behavior setting. These fine-grained descriptions of her embodied actions provide an empirical basis to analyze enactive sense-making. On the one hand, they disclose the affective and emotional experience of perceiving relevant affordances in the environment, and on the other hand, they show the specificity of sensorimotor abilities required to join the setting’s standing pattern of behavior.

Guess what’s happening in *Jubilee Plaza* now? Hint: 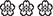
!
Let’s fall in love with everything cherry blossom! Enter the first stop of a romantic cherry blossom world with us by passing through the magical pink walkway at *Jubilee Plaza* AB2! Don’t forget to bring along your BFF to our pop-up store to try on our newest collection and enjoy the sweetness overload selfie moment!
First Stop: March 12–18 at *Jubilee Plaza* AB2
Second Stop: March 25–31 at *Fragrant Mall* 4/F Atrium
(Facebook post, March 13, 2019)

## Introduction

**FIGURE 1:**
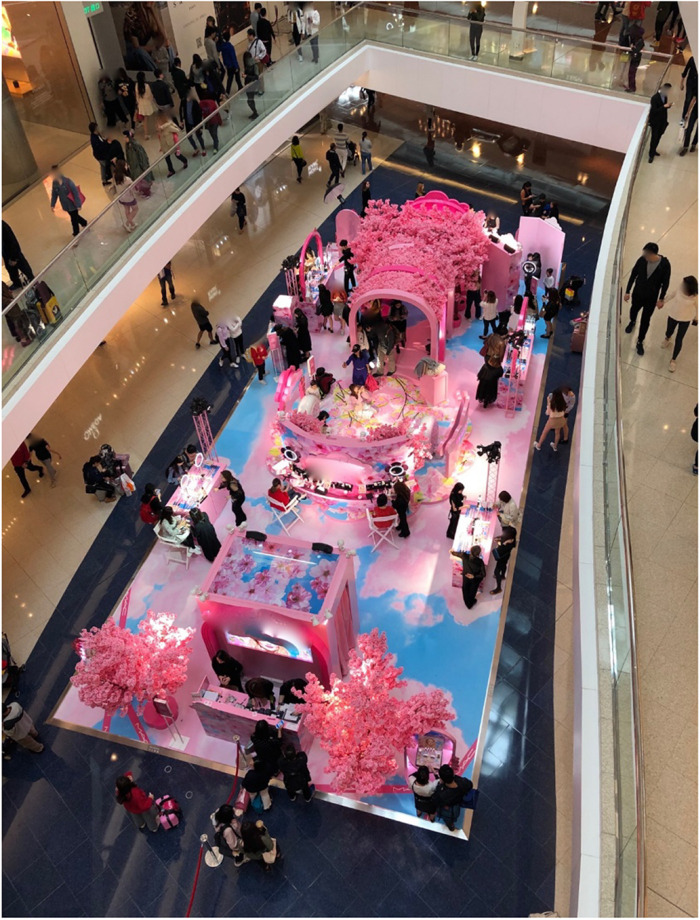
The Pink Cosmetics pop-up store.

On March 13, 2019, a publicly shared post to the official Facebook page of Pink Cosmetics summoned its global community of 20 million followers to Hong Kong’s *Jubilee Plaza* mall.^1^ This giant of the cosmetics industry (annual turnover of 1 billion US$) was inviting Hong Kong customers to join “a romantic cherry blossom world… by passing through the magical pink walkway.” The post encouraged people to come with their friends, enticing them with the opportunity to try on the brand’s latest makeup and take selfies with the “sweetness overload” (a synthetic cherry blossom that could be seen decked around the setting; Figure 1). Several air-brushed images shared with the post provided a bird’s-eye view of an elaborately decorated environment assembled in the mall’s ground floor atrium, along with snapshots of elated inhabitants already interacting, being made up by beauty consultants, and posing for selfies.

The Pink Cosmetics pop-up store sets the scene for this paper’s examination of an intersection between ecological psychology and the enactive approach. Rather than apposing these theories to evaluate their compatibility (see Heft, 2020), this study draws on elements from both—behavior setting theory from ecological psychology (Barker, 1968; Wicker, 1992; Heft, 2018) and enactive-informed approaches to embodied communication (Cuffari and Jensen, 2014; Jensen and Pedersen, 2016; Streeck, 2017)—to conduct a two-pronged analysis of person–environment relations at the pop-up store from complementary levels and perspectives, adopting a specific focus on embodied action and gesture.

The findings first show that embedded in the mall environment is a bounded spatiotemporal structure equipped with several interdependent behavior–milieu parts that invite a range of sense-making behaviors, which are conducive to the application and purchase of makeup. I then examine video recordings of my research participant encountering, entering, and exploring this environment and describe her movements, postures, gestures, and language use as an empirical basis to analyze her sense-making. On the one hand, her embodied actions disclose the affective and emotional experience of perceiving relevant affordances in the environment, and on the other hand, they show the specificity of sensorimotor abilities required to join the setting’s standing pattern of behavior.

In addition to these findings, the study contributes a situated example of how novel forms of consumerism and screen culture are shaping today’s urban environments (Fleming and Harrison, 2020), begging the question “of what kinds of worlds we are building, for whom, and under what constraints and possibilities” (Di Paolo et al., 2018, p. 10).

### Background

Almost 70 years prior to this study, developmental psychologist Roger Barker set up an observational field station at a town in North America called Midwest (pseudonym) to investigate questions that he believed had been wrongly “excised” from psychology: “How do environments select and shape the people who inhabit them? What are the structural and dynamic properties of the environments to which people must adapt?” (Barker, 1968, p. 4).

Barker’s longitudinal study of a town’s inhabitants resulted in the discovery of *behavior settings*, defined as “highly structured, improbable arrangements of objects and events which coerce behavior in accordance with their own dynamic patterning” (Barker, 1968, p. 4). These “extra-individual” patterns of behavior–milieu around the town were shown to be better predictors of his subjects’ behavior (several of the town’s children) than individual psychological attributes or observable sensory inputs. Examples of the town’s behavior settings included a basketball game, a worship service, a piano lesson, and the town’s drugstore during opening hours—each consisting of “one or more standing patterns of behavior-and-milieu” (Barker, 1968, p. 18). Though far removed in time, space, and texture from the environments observed by Barker, the announcement and images of a pop-up store in the *Jubilee Plaza* exhibit the hallmarks of a behavior setting, including information about its occurrence, duration, population, action patterns, and behavior mechanisms (Barker, 1968, pp. 46–80).

Bringing a Gibsonian approach to behavior settings and framing their patterns of behavior–milieu in the language of dynamical systems, Heft refers to behavior settings as “*higher order dynamic units of the environment* constituted by the joint actions of the individuals and the material features (‘milieu’) at some locale” (Heft, 2018, p. 107; emphasis original). Heft has noted that Barker’s “momentous” discoveries remain “under-appreciated” and even “ignored” in the study of human development, behavior, and psychology (Heft, 2003, 2018).^2^ In the embodied cognitive science and philosophy of the mind, by contrast, the theory of behavior settings (and Heft’s dynamical reframing) has been recognized for its potential to complement the enactive approach (McGann, 2014, 2016; Di Paolo et al., 2017, 2018).

The enactive approach refers to a view of embodied cognition that is rooted in the self-organizing principles of biological processes (Varela et al., 1991/2016; De Jaegher and Di Paolo, 2007; Thompson, 2007; Di Paolo et al., 2017, 2018; Gallagher, 2017). According to Evan Thompson, enaction “drew on multiple sources” including but not limited to “the theory of living organisms as self-producing or ‘autopoietic’ systems…,” empirical evidence from embodied cognitive science that “sensorimotor interactions with the world shape cognition,” and concepts from Merleau-Ponty’s phenomenology, such as *intercorporeality* (Varela et al., 1991/2016, p. xxv; Meyer et al., 2017).

Enactivists join ecological psychologists in viewing people and environments as fundamentally intertwined (McGann, 2016). Barker’s behavior settings theory focused on describing structures and dynamics in the environment and their role in selecting and shaping human behavior *en masse*. In contrast, enactivists begin with the self-organizational properties of individual organisms and examine cognitive activity as *sense-making*, defined as the “active adaptive engagement of an autonomous system with its environment in terms of the differential virtual implications for its ongoing form of life” (Di Paolo et al., 2018, p. 332).

In the enactive approach, people and their environments are always already materially enmeshed on a biological level through a ceaseless process of individuation: self-production and self-distinction (Thompson, 2007). As people act and interact with their environment, they regulate the organism–environment coupling relation through the moment-to-moment “selective opening and selective rejection of material flows” (Di Paolo et al., 2018, p. 40), and in doing so, they enact the significance of the world they inhabit with respect to their own vital norms (De Jaegher and Di Paolo, 2007; Cuffari et al., 2015; Di Paolo et al., 2018). “Whatever the organism encounters,” Thompson (2007) reminds us, “it must evaluate from the vantage point established by its self-affirming identity” (p. 154). In this perspective, the environment emerges dynamically from the individual’s sense-making behaviors.

The ecological and enactive views on the person–environment relation embody an ongoing source of tension between the two approaches, with the pre-given (publicly accessible) character of the built up environment “out there” on the one hand, and the claim that individuals enact the significance of the world through sense-making behaviors rooted in the organism’s individuation on the other hand (McGann, 2014, 2016; Heras-Escribano, 2016; Di Paolo et al., 2017). McGann (2014) has explicitly formulated the gaps that each approach consequently sees in the other. In enactive approaches to cognition, “[t]he precise nature of the environment is frequently left ill-described” (McGann, 2014, p. 2), while in ecological psychology, “much of the texture and detail of the agent… in a description of a given engagement” is similarly left wanting (McGann, 2014, p. 3). However, “what is actually happening,” as McGann (2016) later stresses, “is both are giving compatible and possibly even equally valid accounts of things, just at different resolutions of description” (p. 313). A number of researchers have proposed that behavior setting theory in particular could provide a wide-angle lens for considering enactive issues (McGann, 2014, 2016; Heras-Escribano, 2016; Di Paolo et al., 2017, 2018), though specific attempts to do so are lacking.

### Embodied Action and Gesture: Bridging the Ecological/Enactive Divide?

As enactivists have sought to expand their living systems approach to encompass sensorimotor abilities and intersubjectivity, they have pointed to studies of face-to-face interaction in social settings for evidence of sense-making (Gallagher, 2005, 2017; Cuffari et al., 2015; Di Paolo et al., 2017, 2018). However, fine-grained analyses of embodied action, language use, and gesture have not figured prominently in the core enactive literature (though see Gallagher, 2005, 2017).^3^ And yet, the case can be made that strands of embodied communication research are fruitfully bridging the ecological/enactive divide. Micro-ethnographic research into human communication in particular is characterized by rich descriptions of sense-making behaviors analyzed in relation to specific ecological environments and niches (Streeck, 2009, 2017, 2018; Cuffari and Jensen, 2014; Jensen and Pedersen, 2016; Cuffari and Streeck, 2017; Meyer et al., 2017; Goodwin, 2018; Harrison, unpublished).

A case in point is Streeck’s (2017) suggestively titled *Self-Making Man*, a book-length analysis of one man’s behaviors over the course of a day’s work in his automotive garage. Streeck (2017) acknowledges the similarity of his study to the “specimen records” of individual inhabitants encountering behavior settings in Midwest (Barker and Wright, 1951). But as with the current paper, he adopts a micro-ethnographic perspective on the garage owner’s embodied behavior to show how “each moment of understanding is the result of a local montage of heterogeneous resources” (p. xxviii), which range from individual histories to grammatical structures, gestures, and ecological objects on the garage shop floor.

Within this ecological friendly view of person–environment relations, Streeck (2017) also draws on enactive individuation, viewing embodied behavior as self-making (hence the title of his book) and developing an approach to “autopoieses by gesture” (pp. 287–293). Over the course of several chapters, Streeck (2017) shows, for example, “how gestures adapt to settings while structuring them… how gestures’ orientations are embedded in the intercorporeal context of the moment… (and how) gesturing not only varies moment by moment in response to communicative needs or emerging significances of the moment, but also in a broader fashion between occasions” (pp. 288–292). The embodied communicative and meaning-making practices of the garage manager are always analyzed with respect to the significances he perceives and enacts on his shop floor, that is, to the functional and social affordances of a behavior setting (though Streeck does not address the garage shop floor explicitly in light of behavior setting theory).

Adopting a similar analytical methodology to Streeck—which combines transcription of naturally occurring interaction data with analytical notions from cognitive semiotics, ecological psychology, and the enactive approach—Jensen and Pedersen (2016) offer another example of gesture research bridging the ecological/enactive divide. These researchers situate their study of embodied action within clearly identifiable “organizational eco-systems,” including “a hospital, a school for children with special needs, and a kindergarten” (p. 86). Alluding to individuation, they define enactive sense-making simply as “a pull toward certain aspects [of these environments] at the expense of others” (Jensen and Pedersen, 2016, p. 86; see also Thompson, 2007, p. 154; Di Paolo et al., 2018, p. 146). The nature of this pull is shown through a fine-grained study of participants’ embodied actions, gestures, and situated language use, which disclose the emotional and affective attunement of participants to certain affordances in the environment. While the environments were shown to supply “a set of expectations of how specific actions can be carried out by the participants to achieve pre-defined goals” (cf. Barker’s “program circuits”; Barker, 1968, p. 168), the sense-making behaviors of individuals accounted for the unexpected actions, trajectories of situated action, and emergent affordances observable in the data (Jensen and Pedersen, 2016, p. 86).^4^

Building on enactive-informed micro-ethnographic studies of human communication in specific ecological environments, this paper takes the research participant’s encounter with the Pink Cosmetics pop-up store at the *Jubilee Plaza* mall as a case study to demonstrate how concepts from ecological psychology and the enactive approach can be utilized to account for an individual’s actions in a public setting from complementary levels and perspectives. A research question that addresses these different levels and perspectives is: How do people’s sense-making behaviors relate to features of the behavior setting they have joined? Unpacking this question will require a double-pronged analysis of two specific sub-questions: (a) What are the structural and dynamic properties of the behavior setting? And (b) What sense-making behaviors are revealed through the actions of individuals as they join and explore this setting? The next section introduces the data and clarifies the case study methodology.

## Data and Methods

### The Corpus

My recordings and images of interactive episodes in the Pink Cosmetics pop-up store are taken from a video-recorded corpus of everyday life in Chinese settings. Central to this corpus is a Chinese woman who gave informed consent to become a key participant in the research and allowed me to record her in a range of social and professional interactions. In filming, I have aimed to follow a micro-ethnographic approach to human action and interaction, recording with a single hand-held digital camera, guided by conventions in multimodal interaction research (see for example Streeck, 2009, Ch. 2; Streeck, 2017). I have also conducted semi-structured interviews with this participant, as well as think-aloud paradigms and post-event recalls, which I have been able to draw on to support or nuance interpretations of her embodied interaction. While acknowledging the specificity of this corpus, it has also contributed a situated example (Busch-Jensen and Schraube, 2019) for reflecting on contemporary subjectivities, lifestyles, and urban environments in China (Fleming and Harrison, 2020).

### The Case Study

The case study for the current paper was recorded one afternoon in the *Jubilee Plaza* mall, a 1 million-square foot retail space located centrally in Hong Kong. The research participant had been shopping at the mall, when I alerted her to an elaborately decorated installation in the mall’s main atrium and followed her there with the camera. I captured her first perception of what transpired to be the cosmetics pop-up store and continued filming as she approached, entered, and explored. Six video clips were made on this occasion, totaling 9 min 43 s. Some of these clips include moments of short dialogue between myself and the participant.

In addition to this video-recorded corpus and to the time that I spent in the store when recording, my case study draws on various ethnographic materials. These include the store’s own descriptions posted to Facebook, the labels and signs that I observed in the store (both in person and visible in the data), notes taken from many conversations that I have had about this store with the research participant following the data collection, the website of the Jubilee Plaza mall, and finally, my experience of passing through this atrium and shopping in the mall over a 12-month period.

This case study could be considered as “extreme” (Flyvbjerg, 2001), meaning “well suited for getting a point across in an especially dramatic way” (p. 78). According to Flyvbjerg (2001), “extreme case studies often reveal more information because they activate more actors and more basic mechanisms in the situation studied” (p. 78). In terms of a behavior setting, for example, the pop-up store combines an intriguing combination of esthetic, business, education, personal appearance, and social contact action patterns (Barker, 1968, pp. 52–66). In terms of enactive issues, its “romantic,” “magical,” and digital qualities seem contrived to solicit specific emotions and affects, which have been established as important for sense-making and cognition more generally (Jensen and Pedersen, 2016; Gallagher, 2017).

### Procedure of Analysis

To answer the research questions, I adopted a two-pronged analysis. I first applied Barker’s criteria to describe salient aspects of the structure and dynamics of the settings, then examined the video recordings of my key participant exploring this setting to analyze her visible bodily actions as evidence for enactive sense-making.

Barker (1968) developed “three tests for evaluating any part… of a community as a possible behavior setting” (p. 23). The first of these was structural; the second two concern its dynamics. The structural test helps distinguish the pop-up store as a behavior setting, while the dynamic tests reveal behavioral objects, mechanisms, and action patterns that are salient to its internal unity. Barker implemented these tests quantitatively with longitudinal data and multiple raters. I apply his descriptors and definitions to describing the public environment qualitatively.

In the second step of the analysis, I zoom into my participant’s sense-making behaviors by examining the video recordings of her in the setting. I follow the enactive-informed approach to embodied communication developed by Streeck (2017), who identified his research participant’s sense-making behaviors by describing “his habits of walking and standing, of looking and pointing, his methods of showing others how things work…, for gesturing and speaking and organizing” (p. xxix). The analysis involves attention to the environmentally embedded moment-to-moment behaviors visible and audible in the video recordings, such as the person’s “gait and posture, gaze, gesture” as well as analysis of the participant’s language use and conversation (Streeck, 2017, p. xxix). This is an abductive phase of analysis, “a slow procedure, characterized by continuous shifts between a purely descriptive and a theoretical orientation to the data” (Streeck, 2017, p. 390). A similar “analytical movement” between scales of analysis, description, and theory has been proposed in psychology research as a strategy for situated generalization, referred to imagistically as *zooming in zooming out* (Busch-Jensen and Schraube, 2019).

## Analysis

### Pink Cosmetics Pop-Up Store as Behavior Setting: Structure and Dynamics

To be a behavior setting structurally, according to Barker (1968), a part of the community must be a “behavior–milieu synomorph,” meaning to have the following “essential structural characteristics” (p. 38):

(a) a standing pattern of behavior (a bounded pattern of behavior of people *en masse* which occurs independently of the particular persons involved), (b) anchored to a particular milieu complex; (c) at particular time–space loci; (d) with behavior and milieu synomorphic; (e) and with milieu circumjacent to behavior (Barker, 1968, pp. 37–38).

The *Jubilee Plaza* mall is described on its website as “an energized environment of innovation, originality, and pleasure… including over 200 retail stores and restaurants, a multi-screen cinema, one of Hong Kong’s largest ice rinks, (and) over 220,000 square feet of office space.” Like “the churches, the schools, and the courthouse” observed in Midwest by Barker (1968, p. 23), the mall would be a “multiple-setting synomorph.” The individual retail stores, restaurants, cinema, ice rink, and offices, on the other hand, are more likely to exhibit the structural attributes of behavior settings.

Each of the outlets in the mall is anchored to a particular location that opens and closes according to operating hours (attribute *c*). They each involve a “standing pattern” of behavior, meaning the activities among people in that spatiotemporal locale are to some extent interdependent (attribute *a*). In each case, we can identify a unique *milieu* (attribute *b*). As per Heft (2018), the milieu “refers to the features of the environment that support these patterns of action and, for the most part, are synonymous with affordances in a Gibsonian sense” (p. 109; cf. Gibson, 1986). The standing pattern of behavior is closely bounded by and reflected in the structure of the locales they are in (attributes *d* and *e*). Meaning, not only does the spatiotemporal boundary of the individual store provide a boundary for or “enclose/environ” the pattern of behavior observed therein (attribute *e*), but also, following Barker (1968, p. 19), “there is an essential fittingness” between the pattern of behavior and “the fine, interior structure” of the setting (attribute *d*). If any one of these attributes was lacking for the individual stores, they would likely cease to operate. Meanwhile, the mall would continue to function (hence being classed as a “multiple-setting synomorph”).

Before examining the dynamics of the Pink Cosmetics pop-up store (Barker’s other tests for determining a behavior setting), the store’s structural peculiarities in terms of its time–space locale (criteria *c*) and other attributes of behavior settings merit further discussion.

#### The Time–Space Locale, Pressure, and Population of a Behavior Setting

Pop-up stores by definition are temporary both in time and space. According to the online consultancy firm *Storefront*, pop-up stores are located in “high foot-traffic areas” and last for “typically 3 days to 3 months.”^5^ The Facebook post by Pink Cosmetics announced that the pop-up store was “happening now” in “*Jubilee Plaza* AB2” and would last from “March 12–18.” The location “AB2” is an atrium *cum* thoroughfare between the various landmarks surrounding the mall, including an MTR Station and a university.

**FIGURE 2:**
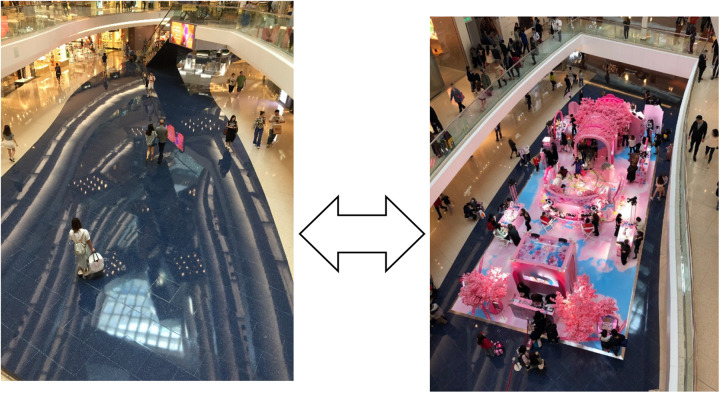
Sky-lit shopping atrium cum thoroughfare qua Cosmetics pop-up store.

Pop-up stores in AB2 are common, but the atrium is often empty too, instead offering its inhabitants a “sky-lit” shopping experience “combining natural light and open space” (Figure 2).

The montage of the open space *cum* thoroughfare with the Pink Cosmetics pop-up store in Figure 2 emphasizes Heft’s (2003) observation that “the same locale can take on quite different functional meanings at different times,” the difference resulting from patterns of collective action that determine the meaning of *place* (Heft, 2003, p. 175).

The location of the pop-up store in the atrium *cum* thoroughfare also illustrates a variable property of behavior settings that Barker called its *pressure*. “Behavior settings differ in the degree to which they bring pressure upon different population subgroups to enter and participate in them” (Barker, 1968, p. 27). As well as being advertised across social media platforms, whose software supplies companies access to potential customers’ newsfeeds, the pop-up stores at *Jubilee Plaza* are literally placed in people’s way (“high foot-traffic areas”).

**FIGURE 3:**
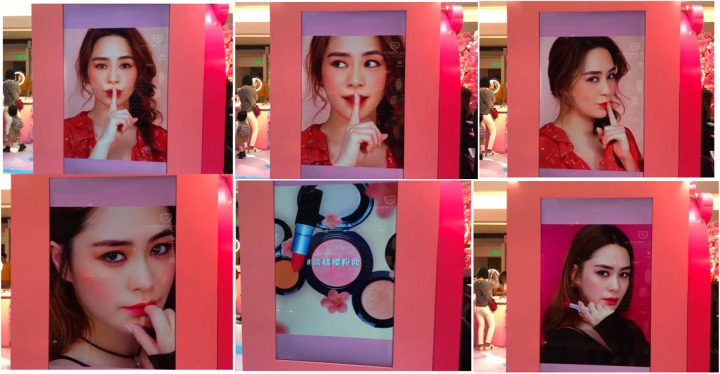
Outward-facing video advertisements targeting subgroup population featuring range of expressive behaviors.

Built into the design of the Pink Cosmetics pop-up store, furthermore, were outward-facing video screens relaying the company’s advertisements, which predictably screened images of female models with seemingly immaculate complexions, alternating with images of the company’s product (Figure 3).

Salient to these larger-than-life images were a range of facial expressions, eye-gaze patterns, postures, and manual gestures that would plausibly interpellate passers-by (the processes by which such signs communicate messages and ideologies are the well-known subject matter of critical multimodal discourse analysis; e.g., Harvey, 2013). The advertisements also work to provide models (literally and figuratively) for how people can look and behave. This part of the pop-up store can be seen as projecting important features of the behaviors invited by the setting, namely, the “established ways of acting… (by) those who have already mastered the craft,” revealing *en passant* the way that such environments take part in “largely unobtrusive and unnoticed disciplining of the body” (Rietveld, 2008, p. 989).

To understand the functional meaning of AB2 when occupied by the Pink Cosmetics pop-up store, we now return to Barker’s criteria for internal and external dynamics.

#### Dynamics of the Pink Pop-Up Store: Interdependencies and Other Attributes

In Barker’s theory, the internal and external dynamics of behavior settings referred to the relations of interdependency between different behavior–milieu parts of a community. For example, Barker identified three behavior–milieu parts of Midwest that were interjacent to the town’s drug store (meaning structurally located within the drugstore): namely, a soda fountain, the pharmacy, and the variety department. The question was whether these behavior–milieu parts were separate behavior settings or parts of the drugstore setting. As Barker noted: “Structurally, they are discrete, but dynamically they are so interdependent in their functioning” (Barker, 1968, p. 22), while also being sufficiently independent from any other settings in the town.

To establish the interdependence between the behavior–milieu parts of a setting, Barker (1968) proposed seven different measures and used rating scales to calculate an interdependency score for a sample of 100 synomorphs in Midwest (pp. 40–46). The results established an empirical basis for identifying “community parts with phenomenal reality and with dynamic significance for behavior” (p. 45). The nature of the Pink Cosmetics pop-up store and my case study methodology preclude collecting the longitudinal observations and quantitative data on which Barker’s interdependence ratings were based. Instead, I will proceed by identifying salient features of the milieu, describing the patterns of behavior that could be observed there and evoking interdependence measures that were salient to understanding how the different behavior–milieu parts were related.

**FIGURE 4:**
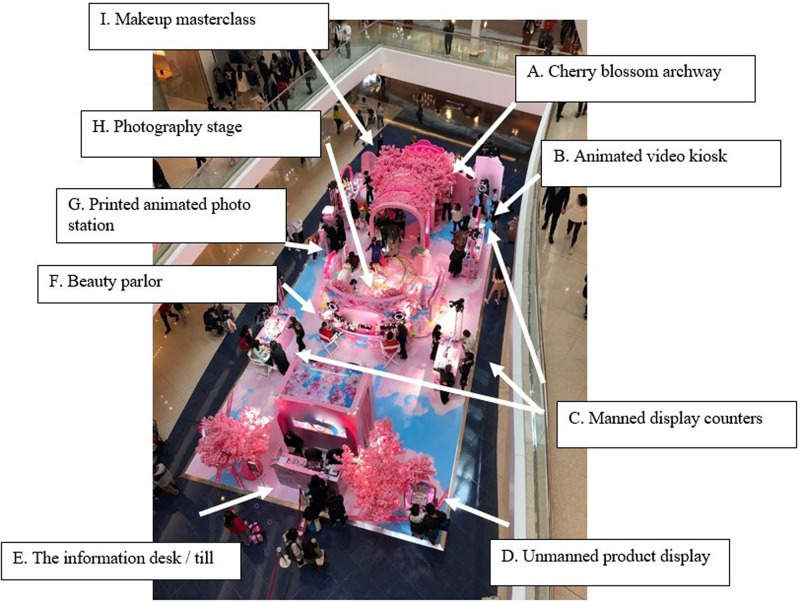
The milieu of the cosmetics pop-up store.

Nine behavior–milieu parts can be clearly identified and labeled inside the pop-up store. While Figure 4 shows a bird’s-eye view of the store’s milieu, the following section will examine this milieu with reference to its corresponding patterns of behavior.

The interdependence measures to be evoked here mainly concern behavioral objects and mechanisms, that is, “the degree to which… the synomorphs use the same or similar behavior objects” and “the same kinds of behavior mechanisms occur in the synomorphs” (Barker, 1968, p. 40). Where salient, an interdependency measure will also be pointed out called “population,” which concerns “the degree to which the same inhabitants enter the synomorphs” (Barker, 1968, p. 40). These observations of the interdependencies are furthermore supplemented with other salient attributes and features described for behavior settings, such as its “action patterns” (Barker, 1968, pp. 52–56).

To start, one set of parts with interdependent behavior objects and mechanisms and where similar action patterns can be observed are the animated video kiosk (Figure 4B), the printed animated photo station (Figure 4G), and the photography stage (Figure 4H). They are each based on the use of screens and image-making technologies, and the associated behaviors they invite are implemented through affective behaviors, gross motor activities, and manipulation. The action patterns relate to esthetics, personal appearance, education, and social contact (Barker, 1968).

**FIGURE 5:**
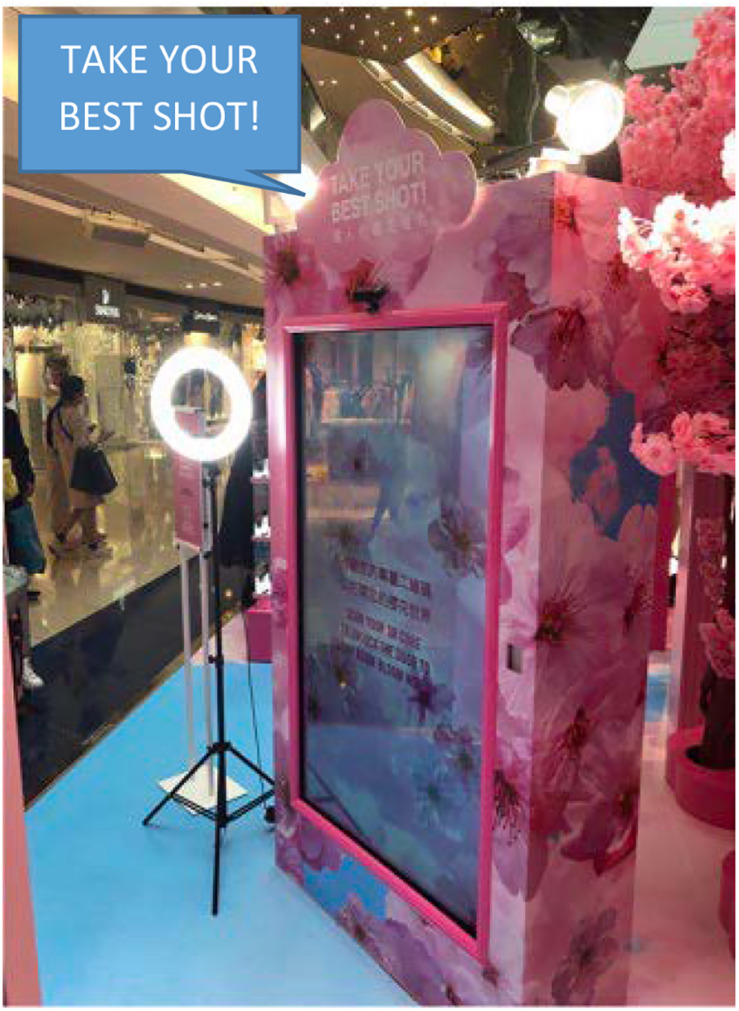
*Take your best shot*! A life-size animated selfie at the video kiosk.

For example, the animated video kiosk (Figure 4B) was a life-size touch screen with a built-in camera on selfie mode illuminated by a tripod-mounted ring light, the screen being encased within a pink cherry blossom frame imploring inhabitants directly to “TAKE YOUR BEST SHOT!” (Figure 5). The kiosk was manned by a beauty consultant tasked with guiding customers in front of the screen, where they could make a video of themselves posing, then evaluate their personal appearance in the draft video. If not satisfied, the users could remake the video of themselves and finally share the finished product online before moving on to the next part or station of the store.

**FIGURE 6:**
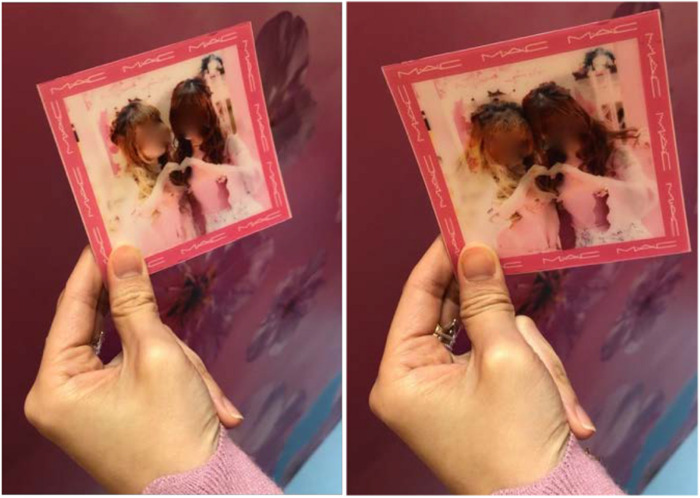
Holographic image from the animated photo station.

A similar behavioral object, mechanism, and action pattern were in place at the printed animated photo station (Figure 4G). Inhabitants of this part of the pop-up store were similarly guided by a beauty consultant in front of a camera, which, rather than making a video, produced a personalized holographic photo. This part of the setting similarly solicited various poses from the inhabitants (such as the very common “hand heart” gesture; Figure 6), which they could evaluate and redo before finalizing the choice of images to be printed. The machine’s algorithm would stitch these images together into one holograph, print this out as a souvenir for the inhabitants and at the same time upload the image online to Instagram. Inhabitants without an Instagram account were prohibited from participating in the standing pattern of behavior associated with this part of the store, which emphasizes the requisite material conditions and desired population targeted by Pink Cosmetics.

In these two parts of the setting, the function of ecological objects was to capture and augment the inhabitant’s self-image to furnish those inhabitants with that image of themselves, who could then evaluate their personal appearance before either redoing the image or sharing it online. In terms of their framing and filtering, as well as the poses they captured, these images were similar to those being relayed in the advertising displayed around the setting (recall Figure 3), creating further ecological unity with other parts of the store.

**FIGURE 7:**
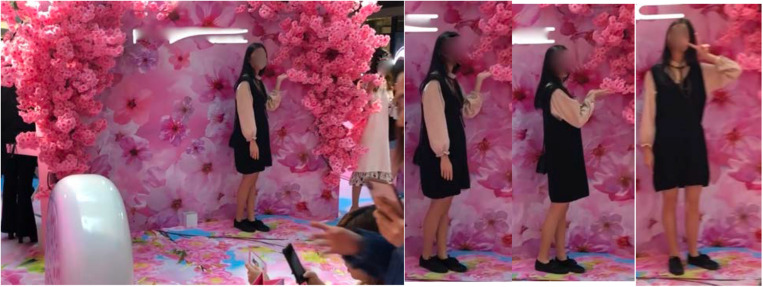
Posing on the photography stage with the cherry bloom.

The third part of the setting which showed clear interdependence with the video kiosk and the animated photo booth was the photography stage (Figure 4H). This was a raised platform mounted with a cherry blossom backdrop and the company’s name in bright neon lights. Though unmanned, signs invited inhabitants to join the stage for an “interactive photo moment”—this being the affective action pattern described by the company on Facebook as to “enjoy the sweetness overload selfie moment” (Figure 7). An example of behaviors associated with this part of the setting can be seen in the sequence of frame grabs, which show one of the setting’s inhabitants performing a series of poses for the camera.

Figure 7 highlights another key behavior mechanism observed by Barker (1968), which was the “involvement of the hands in the standing behavior pattern or setting” (p. 69). Beyond the performance of stock gestures (such as the “V-gesture” and “hand hearts”), posing on the photography stage often incorporated the touching of cherry bloom and other forms of “tactual feeling” and “manipulation,” including sniffing and kissing the fake flowers (for the benefit of the camera). Behavior in other parts of the setting was also implemented through this mechanism wherever synthetic flowers were involved, such as in the cherry blossom archway (a; see section “*Joining the Setting”*).

A functional attribute common to the behaviors discussed so far is what Barker called the “personal appearance action pattern,” defined as “behavior concerned with improving personal appearance *via* clothing, grooming, adornment” (Barker, 1968, p. 61). At the three stations described above, people could be seen engaging in behaviors clearly aimed at improving their personal appearance, such as “looking one’s best” for the camera (Barker, 1968, p. 61). The affordances of the technologies in particular were crucial. The images produced by the different machines would invariably be edited, color corrected, and otherwise filtered to enhance the appearance of the inhabitants and their subsequent appreciation of their own image. When inhabitants used their own cameras (e.g., at the photography stage), they could be seen retroactively photo-shopping their results, which can involve two-dimensional digital skin smoothing, facial feature restructuring, overall slimming, leg lengthening, and teeth whitening procedures.

In relation to this personal appearance action pattern, a rating scale of participation known as “evaluation and appreciation” is highly relevant. This scale refers to “behavior that explicitly recognizes the values of the action pattern, whether good or bad, or tests its effectiveness… To receive a rating, there must usually be a place in the program for appreciation and evaluation” (Barker, 1968, p. 53). All the synomorphs discussed so far included inhabitants appreciating and evaluating their own image. This action pattern was not exclusive to the stations that involved image-capturing digital technology (Figures 4B,G,H) but also occurred at the manned display counters (Figure 4C), where customers could try on tester makeup and evaluate their appearance in brightly lit mirrors.

The application of makeup was obviously a salient gross motor activity at these display counters. Two other settings were similar in this respect, namely, the beauty parlor (Figure 4F) and the makeup master class (Figure 4I). However, the role (or “functional position”) of the consultants across these parts revealed different “degrees of involvement and responsibility” (their “depths of penetration”) with which people could participate in the setting (Barker, 1968, pp. 49–51). While the consultants manning the display stalls typically only offered advice and assistance (as far as I could gather) and cleaned up after the customers, those manning the beauty parlor and the makeup class were trained professionals in the art of makeup application. This reveals an additional pattern being shared across these parts of the setting, namely, education.

Finally, the application of makeup in different parts of the setting reveals further interdependencies between the different stations. Having been made up, inhabitants would then go to take pictures or make videos at other stations. This illustrates both a behavioral and a population interdependence, meaning that “the physical resultants of behavior in A may spread to B and vice versa” and that “people who enter synomorph A also enter synomorph B” (Barker, 1968, p. 41). It is why the inhabitants of the store were likely to cross each other’s paths on various occasions during their time in the store. The application of one’s own makeup was also a frequent behavior of inhabitants in all locations around the store.

With the salient structural features and interdependent dynamics of the pop-up store now sketched, I propose to zoom into the sense-making behaviors of a given individual and examine video recordings of her moment-to-moment perception and exploration of this behavior setting.

### Sense-Making in the Setting

Following Streeck (2017), I will take a description of my participant’s “gait and posture, gaze, gesture” (p. xxix) as well as her situated language use as an empirical basis for reconstructing an account of sense-making behavior, which I understand following Di Paolo et al. (2018) as “cognitive and affective activity manifested experientially as a structure of caring” (p.332). In this approach, Streeck (2017) has shown why any “body motion” is significant because it potentially “enacts the organism’s recognition of a feature of a situation and selects it as significant for the organism” (Streeck, 2017, p. 282; see also Jensen and Pedersen, 2016; Cuffari and Streeck, 2017; Streeck, 2018). “By making gesture,” specifically, it can be seen how “the speaker’s living body orients itself to the cognitive and social landscape at hand as an acting body,” as well as how the speaker “makes sense in the manners in which acting in the material world makes sense” (p. 295). In what follows, these relations between visible embodied action, orientation to significances in the material surroundings, and sense-making will be examined in the footage of our participant encountering the setting (see section “Encountering the behavior setting”), then entering and exploring its different parts (see section “Joining the Setting”).

#### Encountering the Behavior Setting

**FIGURE 8:**
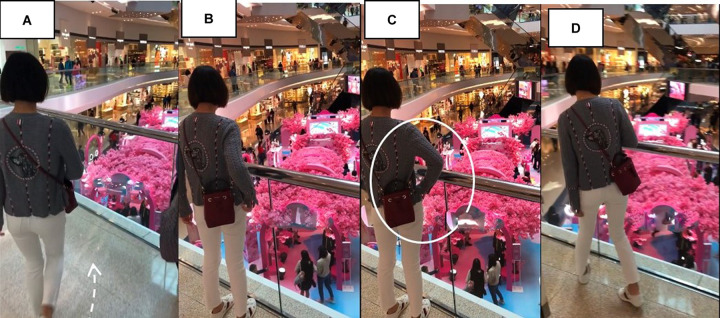
X’s first sight of the pop-up store. **(A)** Approach, **(B)** gazing down, **(C)** hands-on-hips, and **(D)** adjusts position.

X’s first encounter with the pop-up store occurs from a balcony overlooking the mall’s atrium (Figure 8). She approaches this balcony at an assured pace (Figure 8A), spends several seconds gazing down on the setting (Figure 8B), adopts a hands-on-hips posture (Figure 8C), then adjusts her position by moving to her left and craning her neck (Figure 8D).

In these images of X first viewing the pop-up store, we can see her embodied orientation toward the setting. After a forthright approach to the lookout point (Figure 8A), she adopts a hands-on-hips posture or stance (Figure 8C). As Streeck (2018) reminds us, the word stance metaphorically “refers to someone’s disposition toward the situation,” which in the case of a hands-on-hips or “akimbo” posture, has been shown to function in “projecting a sense of non-occupation, an observer’s stance, being at rest” (p. 335). With this possibility in mind, X would seem to spend time taking in her perception of the store. She also adjusts her positioning to a slightly different angle, allowing for different perceptual experiences in which new features of the setting come into view.

**FIGURE 9:**
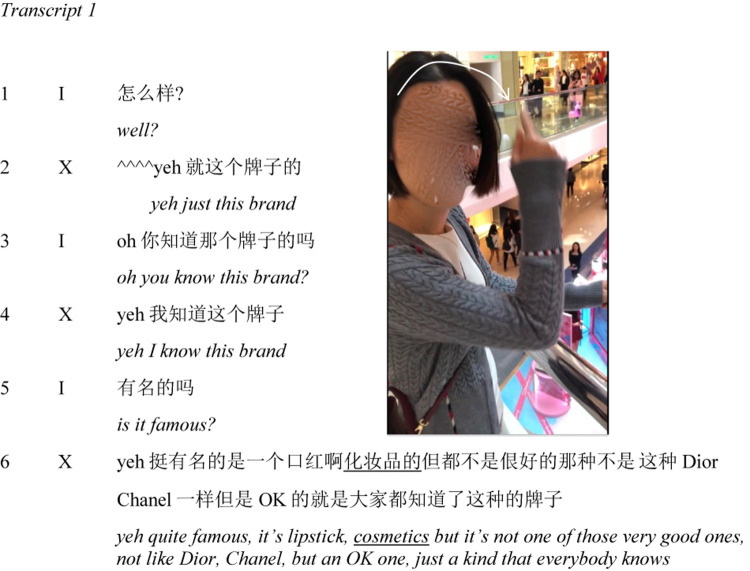
X associates the brand with cosmetics and enacts applying makeup.

After these first 20 s, I engage X in a short dialogue (Transcript 1). I ask her “
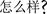
?” (*well?*, line 1). After nodding her head (^^^^), she replies “yeh 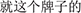
” (*yeh just this brand*, line 2). I check her familiarity with the brand (lines 3–4), then ask about its reputability (line 5). She replies with details about the brand’s offerings and its standing in relation to designer labels (line 6). As she mentions the product “
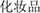
” (*cosmetics*), she makes a gesture across her forehead. The timing of the gesture in relation to speech is indicated with underlining in the verbal transcript, while the gesture is visualized in the accompanying frame grab (Figure 9).

In this short segment, X associates the setting with a brand and with the products it sells. Given the form of her gesture and its timing with the word “
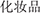
” (*cosmetics*), I interpret this gesture as an enactment of applying makeup. The suggestion is that integral to her initial experience of the store is the relevance of certain embodied actions. Note that while she addresses gaze, speech, and gesture to her addressee, her torso remains oriented or “torqued” toward the setting (Schegloff, 1998). There is a hint of disapproval in her evaluation of the brand as “not one of those very good ones” and “just a kind that everybody knows.”

**FIGURE 10:**
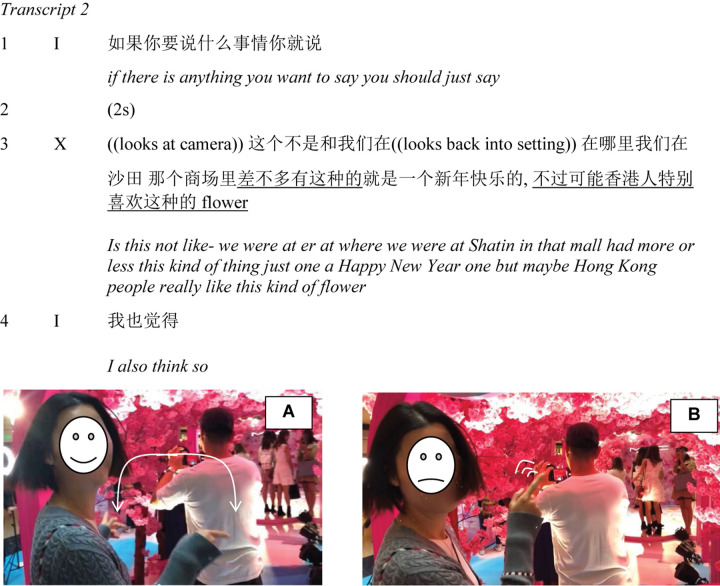
X gestures at the entrance to the archway. **(A)** Tracing the outline of the archway. **(B)** Repeated pointing gestures.

I now follow X down an escalator and toward the pop-up store. At the entrance to the cherry blossom archway, a young couple is taking pictures, so X must wait before entering. As she arrives, I initiate another brief dialogue (Transcript 2). Using a think-aloud protocol, I remind her that if she wants to say anything she should feel free to do so (line 1). After about 2 s (line 2), she evokes a previous trip to a mall in a different part of the city (line 3). Her recollection is initially vague with hesitations and restarts, as she alternates gaze between the cherry blossom archway and the camera (shifts in her gaze direction are indicated in the transcript between double parentheses). When she eventually recalls having seen “
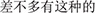
” (*more or less this thing*), her gaze is directed to the camera, she is smiling, but her hands gesture toward the entrance and trace the outline of an arc (Figure 10A). She then expresses the view that “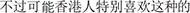 flower” (*maybe Hong Kong people really like this kind of flower*). This is expressed with a slight lean backward, frown, and repeated pointing gestures to the arch (Figure 10B).

**FIGURE 11:**
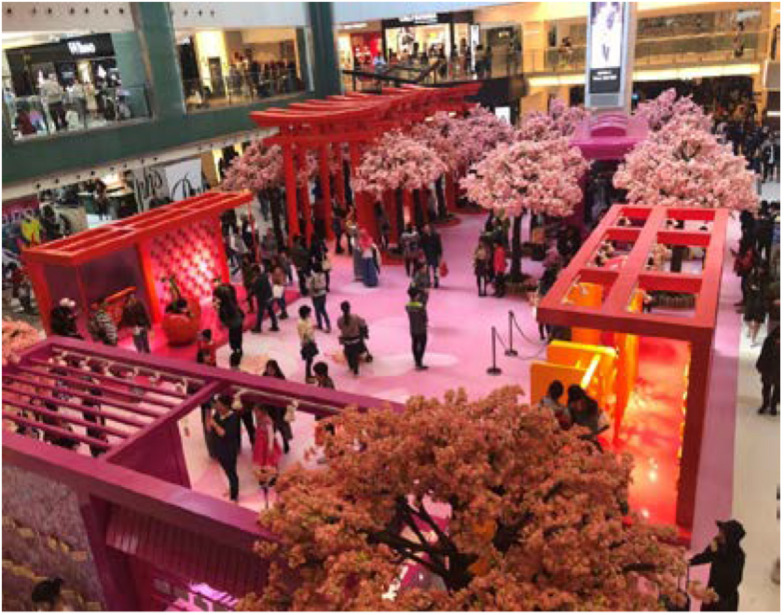
The “Happy Cherry Train Station” display visited by our participant at a different mall in the city.

X’s reference to the “Happy New Year one” at “Shatin” is to a display that she encountered at a different mall a month earlier, where a “Happy Cherry Train Station” had been installed in the mall’s atrium. As reported on the popular blog *Next Stop Hong Kong*, this display came replete with a “Japanese train station, traditional Tori, and beautiful cherry blossom to welcome in the New Year” (Figure 11).^6^

X’s memory of “more or less this thing” indicates her experience of a sense of familiarity recalled from a different time–space locus, central to which is the schematic arch form produced by her gesture. These memories and experiences would be consistent with the experimental findings that features of a behavior–milieu pattern “point to a family of settings” (Heft et al., 2014, p. 389) or to what Barker called a behavior setting “genotype” (p. 80).

Despite experiencing familiarity with the structure of this entrance (specifically its arched form), however, X’s second utterance involves sense-making behaviors that appear to disalign with the setting. Recall that after evoking its similarity, she says “but maybe Hong Kong people really like this kind of flower.” Bearing in mind that pointing gestures can single out referents and transform them into objects of care (Streeck, 2017), her co-occurring frown and backward lean as she makes such gestures, while verbally associating the setting with the preferences of Hong Kongers, indicate a feature of the situation toward which she (as a mainland Chinese) distinguishes herself and does not appear to be favorably inclined (Figure 10B).

The couple in the archway finishes taking photos and proceeds into the store. The archway is empty and becomes available for or “affords” entering by our participant. The next section analyzes her passage through this archway and subsequent exploration of the setting.

#### Joining the Setting

**FIGURE 12:**
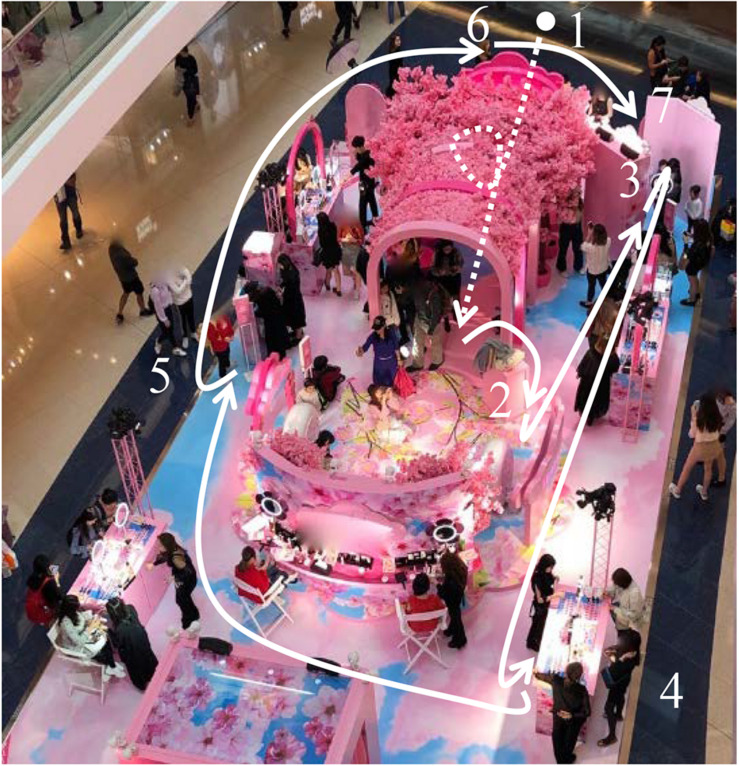
X’s path through the store and the synomorphs she visited.

An overview of our participant’s exploration of the setting is offered in Figure 12, which shows her path through the store with white arrows, reconstructed from our video recording and notes. This figure reproduces the milieu, which, thanks to the behavior setting study, we know to be a set of interdependent behavior–milieu parts (see section 3.1.2.1 above). As can be seen from each point of the white arrow, X’s path brought her to (1) the cherry blossom archway, (2) the photography stage, (3) the animated video kiosk, (4) the manned display counters, (5) the printed animated photo station, back to (6) the cherry blossom archway, and, upon my request, (7) back to the animated video kiosk.

In addition to showing the research participant’s trajectory around the store, this simple reconstruction of X’s path illustrates the population interdependency that was discussed earlier, offering an example of the high degree to which this inhabitant entered different parts of the setting.

**FIGURE 13:**
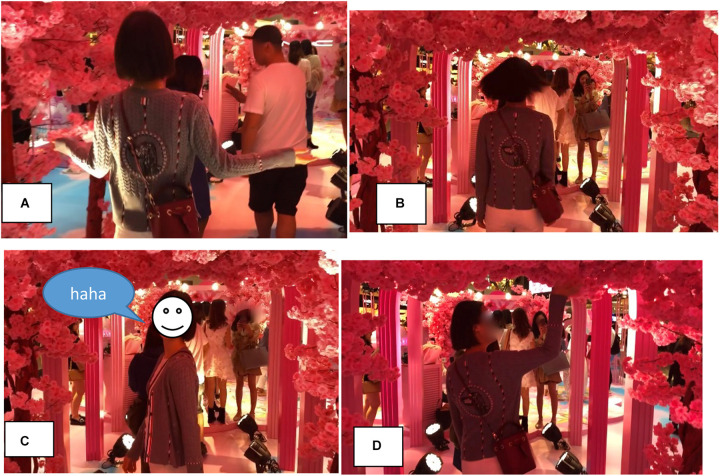
Entering the cherry blossom archway (00:31–00:35). **(A)** Enters, **(B)** pirouettes, **(C)** poses and giggles, and **(D)** fondles and exits.

The archway is decked out with pink cherry blossom and lit up by several powerful floor lights. As shown in Figure 13, when X enters, she extends her arms, skimming the blossom with her fingers (Figure 13A), then spins around to face the camera and giggles (Figures 13B,C). She fondles a roof flower on her way out (Figure 13D).

X’s embodied action as she enters the cherry blossom archway is remarkable and strikingly different to both the assured pace with which she first approached the setting (cf. Figure 8A) and her explicit reflections on the setting while waiting to enter (Transcript 2 and Figures 10A,B). For one, she has slowed her pace down to a leisurely gait or saunter. She seems to be no longer walking to get somewhere or waiting to enter, but partaking in a “passage through the archway,” meaning exploring the material features of this new environment, responding to and engaging with its affordances. Her hands skim the bloom with each step, which is left twitching as she moves by. Two seconds after entering the archway, X pirouettes, smiles, and audibly giggles for the camera (Figures 13B,C), appearing to delight in the experience.

**FIGURE 14:**
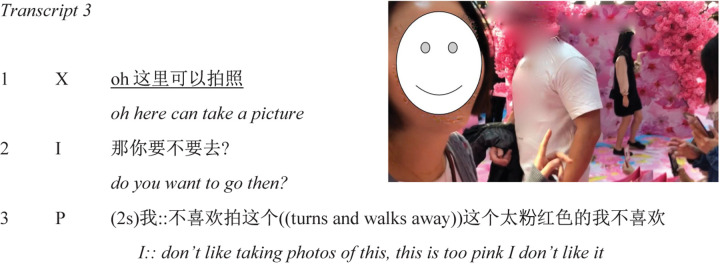
Arrival at the photography stage (00:46–01:00).

X emerges from the archway into a congested space where a group of three women are queuing in front of the photography stage. She compensates, doubles back, and finds an alternative route, navigating the store’s infrastructure to a place where she can view the stage. Having established a clear view on this stage, she momentarily stands still and watches a woman posing for photographs. She then turns to the camera and initiates a dialogue (Transcript 3). Smiling, she points at the stage where the woman is posing for pictures (Figure 14) and says “oh
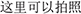
” (*oh here we can take a picture*). I ask her if she would like to take a picture (line 2). After a pause (2 s), she says: “
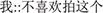
” (*I: don’t like taking photos of this*; where the “::” symbols indicate prolongation of the vowel), then turning and walking away from the stage (and visibly no longer smiling), she says “
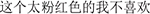
” (*this is too pink, I don’t like it*) (line 3).

This short interchange sheds light on further sense-making behaviors. For instance, conversation analytical work has shown that the linguistic marker “oh” can indicate “that its producer has undergone some kind of change in his or her locally current state of knowledge, information, orientation, or awareness” (Heritage, 1984, p. 299). This would seem to be a coherent interpretation of the current usage, given that X produced her “oh” after a period of silently observing the setting. Judging by her smile and the upbeat prosodic contour of her utterance, the change of state displayed by “oh” seems to have been experienced as a pleasant one. I was wrong to assume X would want to take part in the activity proposed by this part of the setting though. Based on the linguistic and embodied details of X’s response, my offer to take a photograph seems to have triggered quite a negative emotional outburst. In addition to the repeated, direct rejections, by pausing and adding contrastive stress to the pronoun “I” (elongated vowels are attested stress markers in Mandarin conversation; Li, 2014), X resists the assumption implied by my suggestion. As with X’s backward lean, pointing gestures, and frown at the entrance to the cherry blossom archway, the linguistic design of X’s response and her subsequent walking away are behaviors with which she regulates her ongoing relation to (and distinguishes herself from) the immediate environment.^7^

**FIGURE 15:**
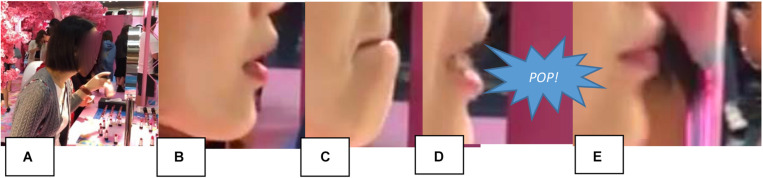
Evaluating lipstick application at display stall (01:44). **(A)** Approach, **(B)** open, **(C)** pinch, **(D)** pop, and **(E)** purse.

After a quick look into the animated video kiosk (where we will return later), X arrives at one of the stalls displaying the store’s product range of cosmetics. Recall that these stalls are equipped with brightly lit mirrors and manned by beauty consultants. In the video, we see several people around the stall using the mirrors to apply lipstick, which they have selected from a range of testers. When a space frees up, our participant approaches the stall, scans the product display, then looks into one of these mirrors for a duration of 4 s. Slowing the video of this mirror-looking episode down, a smooth sequence of actions can be identified. Figure 15 shows how X first cranes her neck to bring her face within 6 inches of the mirror (Figure 15A), opens mouth (Figure 15B), purses lips, uses bottom lip to push the top lip up, uses the bottom lip to pull the top lip down (Figure 15C), which she releases with an audible pop (Figure 15D), then retracts from the mirror with a final lip purse (Figure 15E) before continuing to browse the product display.

The participant’s evaluation of lipstick application at the display stall invites a discussion of the interplay between functional and affective aspects of sense-making behaviors. Recalling the oft-quoted passage on “double sensations” from Merleau-Ponty (1945/2012, p. 95) and Lehtinen (2014) has discussed the lips “as an alternative to the figure of the two hands in the double touch” (p. 80)^8^. While the hands in double touch are often “accounted for in terms of intentionality of conscious acts” and “operative intentionality (the ‘I can’),” Lehtinen (2014) argues that the mode of experience of the lips in double touch “belongs instead to the relations of enjoyment and affective perception” (p. 80). The addition of lipstick and a mirror at the pop-up store problematizes this distinction, however, as we clearly see a task-directed use of the lips to evaluate and improve the appearance of lipstick, which is part of a sociocultural practice. That said, such a functional explanation would leave parts of this action or “sensorimotor scheme” underdetermined (Di Paolo et al., 2017) because X’s lip-smack (*pop!*) seems functionally unnecessary. Unless it is a biomechanical consequence of moving the lips in that way, the lip-smacking might index the relations of enjoyment and affective perception mentioned by Lehtinen. It could also simply mark the ending of the episode. After this pop, X seems satisfied with the appearance of her lips and returns to browsing the products on display.^9^

**FIGURE 16:**
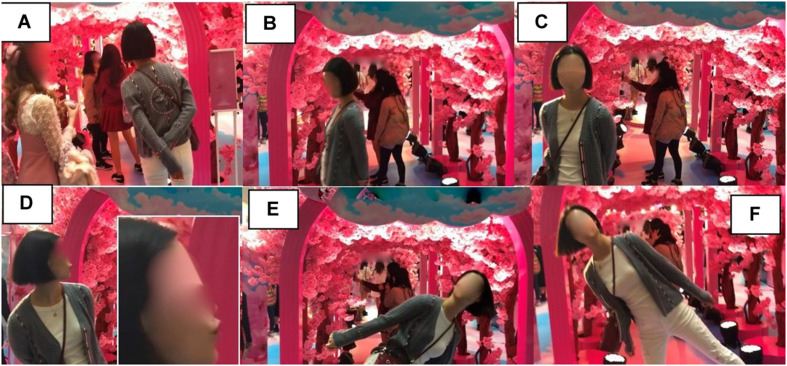
Full-bodied leaning-sideways gesture in front of the archway (05:36–05:48). **(A)** Peering in, **(B)** walking away, **(C)** eye contact with camera, **(D)** posture and pucker, **(E)** lean left, and **(F)** lean right.

After spending a few minutes at the cosmetics display stall, X continues to explore the store and eventually arrives back in front of the cherry blossom archway, which is somewhat congested (Figure 16). Rather than entering again, X stops and peers in. Her hands are clasped behind her back, and she is craning slightly toward the archway (Figure 16A). As the congestion begins to clear, X starts to walk away (Figure 16B). But in doing so, she makes mutual gaze with the camera (Figure 16C). She stops, turns her gaze back to the archway, leans away from it, and begins to pucker her lips (Figure 16D). She then leans the full body first to her left (Figure 16E) then to her right (Figure 16F), each time raising a foot off the floor and extending her arms slightly.

Our participant’s return to the cherry blossom archway is revealing. When seen for the second time, the cherry blossom archway presumably still affords the action of entering, but X does not engage with that affordance on this revisit. Instead, she first adopts another posture that projects an observer stance (Streeck, 2018). Although she then starts to walk away, as she makes eye gaze with the camera, she abandons her trajectory and begins to exploit the archway as the backdrop for a photo opportunity. In doing so, X reveals her perception of an affordance in her own creative way, which triggers an unexpected act (Jensen and Pedersen, 2016).

As for the posing action itself, X’s full-bodied, leaning-sideways-with-pout gesture is very common among the poses for photos in China, belonging to a number of stock gestures that get reproduced on such occasions. According to the research participant X, her leaning-sideways pose imitates the posture of a waddling duck and is supposed to be “cute.” Puckering the lips with this gesture is also according to her “just being cute,” but could be recognized as a manifestation of the more conventionalized “duck-face” gesture or meme.^10^

**FIGURE 17:**
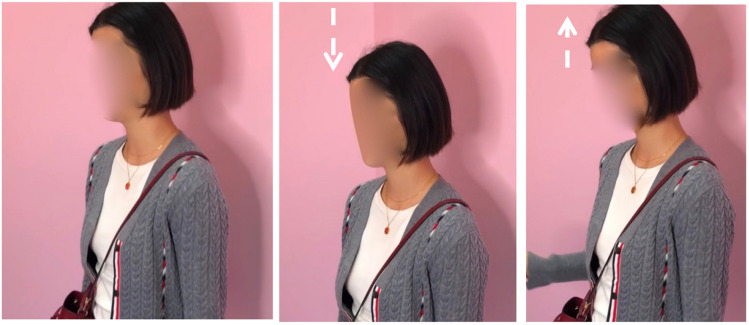
X makes herself smaller to fit on the screen.

Before leaving the store, I ask X to return to the animated video kiosk. After a short wait, the beauty consultant manning the kiosk invites X in. Immediately upon entering, X encounters herself on the big screen. After a moment’s inspection, she says “
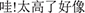
” (*wow! too tall it seems*), and smiling, dips to put herself in the frame (Figure 17).

**FIGURE 18:**
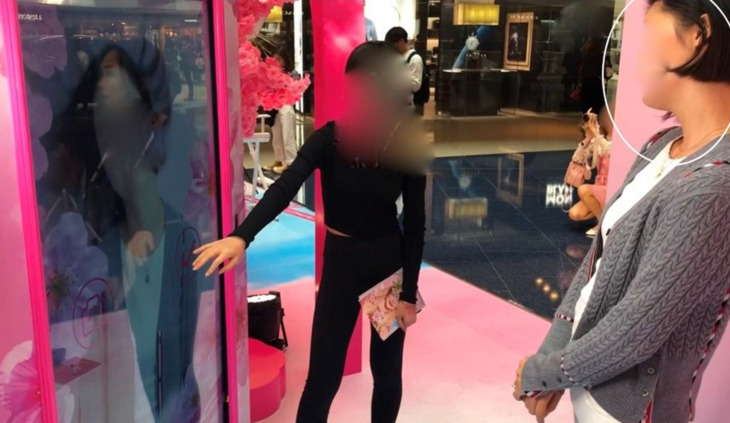
X watching herself, smiles and tilts her head (05:36–05:48).

As a marker of assessment (Schegloff, 1982, p. 85), X’s exclamation “
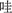
” (*wow!*) is further evidence that she has been invited to assess her appearance, with which she seems astonished. Her dip is an immediate solution: “expert craftsmen will normally immediately distinguish the relevant possibilities for action in situation within their familiar practices” (Rietveld, 2008, p. 981). The beauty consultant now counts X down “Three, two, one,” and as the camera begins recording, X does a hair-flip dance that is well-known on 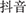 (Chinese TikTok). X’s performance is automatically replayed on the screen. As she watches herself, she starts to smile and tilts her head (Figure 18).

**FIGURE 19:**
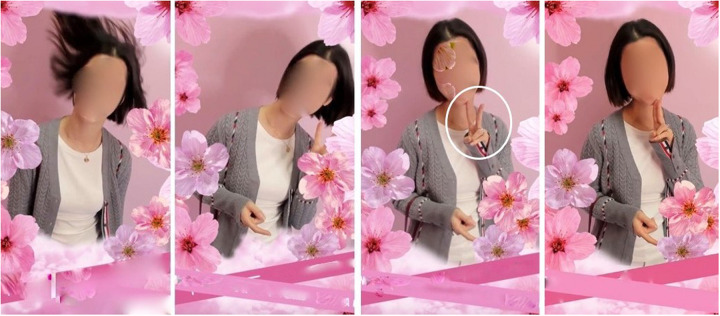
The video sent to the participant’s social media account.

X’s smile and head tilt while watching herself dance can be interpreted as affect-laden sense-making behaviors. “Holding the head in a lateral tilt, or head-cock,” writes semiotician Calbris (1990), “is a sign of tenderness, arising from fondness or from a desire to be touching” (p. 55). Calbris (1990, p. 55) also offers evidence that the gesture is used when “indicating a particular point of view.” X’s smile suggests she is pleased by this view, but she nevertheless does a rerun and includes a “V” gesture at the end of her dance. Interestingly, this V gesture is the same gesture that X observed being performed by somebody posing on the photography stage. This V gesture is drawn from the same class of “cute” stock gestures as the “shush” gesture by the model in the advertisement being projected on the screens outside the store (cf. Figure 3). X’s rerun dance and added gesture can be seen in the post-processed video, which has also been edited, enhanced, and framed with the corporate logo (Figure 19).

As the research participant exits this video kiosk and heads away from the store, leaving the behavior setting, her smartphone pings. An e-mail from Pink Cosmetics has arrived containing her video and a link to the company’s shopping platform online.

## Discussion

This paper has examined an intersection between ecological psychology and the enactive approach brought about by investigating embodied action as sense-making within the confines of a behavior setting. With a case study selected from a video corpus being built to study embodied interaction and urban environments in China (see Fleming and Harrison, 2018, 2020; Harrison and Fleming, 2019), I first described a Pink Cosmetics pop-up store in a Hong Kong shopping mall with the definitions and criteria from behavior setting theory (Barker, 1968). My findings revealed a highly structured environment embedded within the mall, where several behavior–milieu parts (Barker’s “synomorphs”) were interwoven with image-making technologies, including a photography stage, brightly lit mirrors, a printed holograph machine, and an animated video kiosk. Manned by beauty consultants and makeup artists, these machines invariably functioned to capture and augment a person’s self-image, inviting the store’s inhabitants to behave and act by posing, evaluating, and improving their appearance in ways conducive to the application and purchase of makeup.

Having used Barker’s (1968) theory to establish the pop-up store as a behavior setting (describing its structural attributes and identifying salient measures of interdependency between its behavior–milieu parts), I zoomed in to the analysis of a person’s moment-to-moment discovery and perception of this setting. In previous experimental research, people were found to perceive the identity of behavior settings (e.g., a bank, library, basketball game) based on schematic animations of activity patterns shown to them on a computer screen in the lab setting (Heft et al., 2014). For my perception study of the Pink Cosmetics pop-up store, I analyzed visible embodied actions in the actual setting of my key participant, who I had followed at close range with a video recorder as she first encountered then joined the environment of the pop-up store. Adopting a micro-ethnographic approach to embodied communication that draws on both ecological psychology and enaction theory (Jensen and Pedersen, 2016; Streeck, 2017, 2018), I described this person’s gait, posture, eye gaze, gesture, and situated language use as she explored different parts of the store, taking this descriptive work as an empirical basis to identify and analyze her sense-making.

Analysis of this participant’s embodied activity revealed the range of thoughts, feelings, memories, and attitudes that she experienced upon encountering the setting. These included a series of multimodal utterances with which she (a) identified the product on sale (“*lipstick, cosmetics*”) and its associated action patterns (gesturing the application of makeup), (b) judged the store’s brand (“*not one of those very good ones*”), (c) experienced feelings of familiarity from the settings in a different time–space locale (“*more or less this kind of thing*,” the word “this” being accompanied by a gesture singling out a salient design feature of the setting), and (d) evoked cultural explanations for the appearance of the setting (“*maybe Hong Kong people really like this*”), while her facial expression and gestures marked distinctions from her own preferences. As she then entered and toured the store, her visible bodily action (including language) manifested a roller-coaster ride of dispositions and emotions, ranging from disapproval and aversion to amusement, surprise, delight, then dismay and astonishment. Gallagher (2017) stresses that “one’s beliefs and values, as well as one’s affective states and cultural perspective… can shape the way that one quite literally sees the world” (p. 19). By extension, the sense-making activities of an individual—embodied micro-adaptations in relation to the ecological environment that are dependent on her values, beliefs, history, affect, and culture—showed how this individual perceived and experienced the behavior setting studied here.

Focusing on activities occurring in relation to specific structural parts of the setting (what I referred to as “stations”) and moving beyond the analysis of the participant’s multimodal utterances, several patterns of behavior in the data could be identified that involved more kinesically complex sequences of embodied actions patterned in relation to structures in the environment. These occurred more specifically in response to the activities invited by different behavior–milieu parts of the store (its “interjacent synomorphs”; Barker, 1968) and can be seen to highlight further relations between sense-making activities and the specifics of a behavior setting.

Several such behaviors occurred upon encountering different stations in the setting. In addition to behaviors such as standing in line, rerouting upon encountering a congested space, and scanning the display stalls, we also saw the participant twirling through the cherry blossom archway, remaking her lipstick in the brightly lit mirror (*pop!*), and gesturing/dancing for the different cameras (full-bodied leaning, head tilts, hair toss, V gesture, etc.). Perceiving such behaviors as sensorimotor schemes (Di Paolo et al., 2017), enactivists attribute them to “a rich repertoire of ready-made, highly organized ways of engaging the world” (p. 81) that people bring to a behavior setting, or rather will begin to *enact* “when coupled to the right environmental circumstances” (p. 82). While some sensorimotor schemes in the current data “are rather widespread across the species” and across different environments, such as our participant’s standing in line, back-tracking, etc., “others are acquired as part of our sociocultural milieu,” which would be the case for the archway-twirling, posing, and evaluating/improving of one’s appearance invited by this setting (Di Paolo et al., 2017, p. 81).^11^

The sensorimotor schemes which are most closely related to the sociocultural milieu seem crucial to understanding the sense-making/behavior setting relation. They highlight what Rietveld (2008) describes, following Merleau-Ponty (1945/2012), as the participant’s “unreflective performance” or “form of *embodied* intelligence or cognition that is ‘motivated’ by the situation…” (p. 975). Rietveld’s (2008) account of embodied, situated, and lived normativity helps to recognize actions such as twirling through the cherry blossom archway, remaking lipstick in the brightly lit mirror, and gesturing/dancing for the different cameras as the participant’s adequate perception, recognition of, and response to the settings relevant functional affordances. Her “unreflective” performance of these actions also points to the overall (or interdependent) “*potentiating*” and “*affective allure*” of the setting’s structure and dynamics (p. 977). The behavioral objects, mechanisms, and action patterns in this particular setting (which were shown to be based around the making, evaluating, appreciating, and enhancing of self-image) exaggerate both the normative dimension of sense-making actions within a behavior setting and the embodied know-how required of inhabitants to fully join or “penetrate” a setting’s standing pattern of behavior.

Finally, this case study shows what specialists in the design and implementation of pop-up stores like those in *Jubilee Plaza* must already know: “Retail is no longer about buying products but rather it’s about providing an experience that consumers delight in”^12^. Notwithstanding the limitations of my case study, I offer the Pink Cosmetics pop-up store as an example of the cognitive and technological environments being shaped by novel forms of consumerism and screen culture and the sociocultural specificity of the sense-making behaviors that such environments invite. If Roger Barker and the inhabitants of 1950s Midwest were suddenly introduced to the behavior setting studied here, we might wonder how their sense-making behaviors would differ.

## Data Availability Statement

The datasets generated for this study will not be made publicly available. The video recordings on which the analysis is based contain material that would be inappropriate to make open access.

## Ethics Statement

The study of video recorded interaction sampled here was reviewed and approved by the University of Nottingham Ningbo China. The research participant gave informed consent.

## Author Contributions

The author confirms being the sole contributor of this work and has approved it for publication.

## Conflict of Interest

The author declares that the research was conducted in the absence of any commercial or financial relationships that could be construed as a potential conflict of interest.

## Footnotes

1 Details have been modified to preserve anonymity.

2 This apparent neglect is despite behavior setting theory being carried forward and developed by Barker’s former students, such as Schoggen (1989) and Wicker (1992).

3 In *How the body shapes the mind*, Gallagher (2005) reports lab studies of gesture during solicited narratives in a highly controlled lab setting (pp. 107–129), while in *Enactivist interventions* (Gallagher, 2017), he leverages studies of gesture in naturally occurring, multi-party situated activity (pp. 156–158). The differences between these two data sets and the conclusions to which they lead Gallagher are striking.

4 There are overlaps between the interplay of sense-making behaviors and organizational structures in the wider environmental setting in these gesture studies with approaches formulated elsewhere as the “enactive affordance-based model” (de Haan et al., 2013) and a “user-based account of information” in the environment (Withagen, 2018). For more theoretical discussions of such, see also Rietveld (2008) and Rietveld and Kirverstein (2014).

5 https://www.thestorefront.com/mag/what-exactly-is-a-pop-up-shop/

6 https://www.nextstophongkong.com/hong-kong-chinese-new-year-shopping-mall-display-decoration/#new_town_plaza

7 I am grateful to multimodal interaction researcher Xiaoting Li for discussing the morphosyntactic, phonetic, and interactive details of this example with me.

8 Lehtinen (2014) is introducing the work of Luce Irigaray’s phenomenology of the feminine being.

9 An affective reading of X’s lip-smack would be supported by the popularity of lip-smacking in autonomous sensory meridian response videos on YouTube. Furthermore, when I showed this clip to the research participant and asked her what she was doing, she replied “if you don’t know, it is because you have never worn lipstick.” Her response recalls Wittgenstein’s *reason for not having a reason*: “This is simply what I do” (cited in Rietveld, 2008, p. 983).

10 For a discussion of this leaning-sideways pose, I am grateful to Enhua Guo, who helped me to informally survey several Chinese women for their opinion on its meaning. None of these women mentioned the pose being related to ducks, however. Summarizing their views, the pose might have originated with a Chinese Internet celebrity, whose attractive or cute performance of the pose began circulating online, which led to the pose going viral. For a discussion of how gestures are embedded in global cultural and capitalist flows, as would be the case in the uptake of a celebrity pose, readers are pointed toward chapter 4 of Fleming and Harrison (2020) where they will find analyses of a K-Pop dance move appearing in the classroom of a British university campus in China.

11 In addition to species-wide and sociocultural, Di Paolo et al. (2017) state that other sensorimotor schemes are idiosyncratic. The point is that “all of them together reflect the history of each particular body” that enters a given behavior setting (p. 81).

12 See www.storefront.com. Storefront markets itself as “the world’s largest marketplace to book short-term retail space” https://www.whub.io/startups/storefront.
